# Three-dimensional structure of β-cell-specific zinc transporter, ZnT-8, predicted from the type 2 diabetes-associated gene variant SLC30A8 R325W

**DOI:** 10.1186/1758-5996-2-33

**Published:** 2010-06-05

**Authors:** Rob NM Weijers

**Affiliations:** 1 Teaching Hospital OLVG, Onze Lieve Vrouwe Gasthuis, Amsterdam, The Netherlands

## Abstract

**Background:**

We examined the effects of the R325W mutation on the three-dimensional (3D) structure of the β-cell-specific Zn^2+ ^(zinc) transporter ZnT-8.

**Methods:**

A model of the C-terminal domain of the human ZnT-8 protein was generated by homology modeling based on the known crystal structure of the *Escherichia coli *(*E. coli*) zinc transporter YiiP at 3.8 Å resolution.

**Results:**

The homodimer ZnT-8 protein structure exists as a Y-shaped architecture with Arg325 located at the ultimate bottom of this motif at approximately 13.5 Å from the transmembrane domain juncture. The C-terminal domain sequences of the human ZnT-8 protein and the *E. coli *zinc transporter YiiP share 12.3% identical and 39.5% homologous residues resulting in an overall homology of 51.8%. Validation statistics of the homology model showed a reasonable quality of the model. The C-terminal domain exhibited an αββαβ fold with Arg325 as the penultimate N-terminal residue of the α2-helix. The side chains of both Arg325 and Trp325 point away from the interface with the other monomer, whereas the ε-NH_3_^+ ^group of Arg325 is predicted to form an ionic interaction with the β-COO^- ^group of Asp326 as well as Asp295. An amino acid alignment of the β2-α2 C-terminal loop domain revealed a variety of neutral amino acids at position 325 of different ZnT-8 proteins.

**Conclusions:**

Our validated homology models predict that both Arg325 and Trp325, amino acids with a helix-forming behavior, and penultimate N-terminal residues in the α2-helix of the C-terminal domain, are shielded by the planar surface of the three cytoplasmic β-strands and hence unable to affect the sensing capacity of the C-terminal domain. Moreover, the amino acid residue at position 325 is too far removed from the docking and transporter parts of ZnT-8 to affect their local protein conformations. These data indicate that the inherited R325W abnormality in SLC30A8 may be tolerated and results in adequate zinc transfer to the correct sites in the pancreatic islet cells and are consistent with the observation that the *SLC30A8 *gene variant R325W has a low predicted value for future type 2 diabetes at population-based level.

## Background

This report continues our analyses of the genetic factors playing an important role in the pathogenesis of type 2 diabetes [1]. Genome-wide association studies have currently identified single nucleotide polymorphisms (SNPs) within up to 10 genes associated with an increased risk of type 2 diabetes [2-6]. Several of the SNPs identified within or near these genes are hypothesized to influence β-cell function. Previous studies of the latter genes additionally identified the SNP rs13266634 as a nonsynonymous SNP causing an arginine to tryptophan change at position 325 (R325W) in the last exon of the solute carrier family 30 (zinc transporter; ZnT) member 8 (*SLC30A8*) gene on 8q24 (Table 1). Yet, contrary to the outcomes of the above-mentioned association studies, combining the genetic variants including the *SLC30A8 *gene variant R325W was recently reported to have low predicted value for future type 2 diabetes at population-based level [7-10]. From a different point of view, we continued our analyses to have informed discussion, and studied at atomic level the impact of the R325W mutation on ZnT-8 complete with sensor, actuator and transporter parts.

**Table 1:** Overview of confirmed type 2 diabetes association results in the combined stage 1 and 2 samples for the widely replicated type 2 diabetes-associated variant SLC30A8 R325W (rs13266634).

Study	Total sample size; stage 1+2 (number of cases/controls)	OR (95% CI)	*P*-value	**Ref**.

DGI	13,781 (6,529/7,252)	1.07 (1.00-1.16)	0.047	2
WTCCC/UKT2D	13,965 (5,681/8,284)	1.12 (1.05-1.18)	7.0 × 10^-5^	3
FUSION	4,808 (2,376/2,432)	1.18 (1.09-1.29)	7.0 × 10^-5^	4
DGI-WTCCC/UKT2D- FUSION (all data)	32,554 (14,586/17,968)	1.12 (1.07-1.16)	5.3 × 10^-8^	2-4
Icelandic case control*	16,398 (3,836/12,562)	1.15 (1.08-1.22)	3.3 × 10^-6^	5
France case control	5,511 (2,617/2,894)	1.18 (0.93-1.43)† 1.53 (1.22-1.84)‡	6.1 × 10^-9^	6

*Combined European ancestry groups. †Heterozygous. ‡Homozygous.

The *SLC30A8 *gene encodes a 369-amino acid protein, ZnT-8, that transports Zn^2+ ^(zinc) from the cytoplasm into insulin secretory vesicles, where insulin is stored as a hexamer bound with two zinc ions before secretion [11-14]. The ZnT-8 protein is specifically expressed in pancreatic β-cells and thus may be of primary importance for the insulin secretory pathway. Variations in SLC30A8 may affect zinc accumulation in insulin granules, affecting insulin stability, storage, or secretion.

In mammalian cells, eight homologous zinc export proteins, named ZnT-1 to -8, have been described [11,14]. These proteins are members of the SLC30 subfamily of the cation diffusion facilitator family. Analysis of genome sequences shows that cation diffusion facilitators represent a ubiquitous protein family, encompassing more than 400 evolutionarily related members found in species ranging from bacteria and yeast to plants and mammals [15-17]. This protein family is characterized by an N-terminal hydrophobic domain in addition to a C-terminal cytosolic, hydrophilic region that is highly variable both in sequence and in length [18] with a common and remarkably evolutionarily conserved αββαβ fold. Despite the relatively low sequence homology in the cytoplasmic domains, the structural homology between them is impressive. For example, the αββα structural core of the C-terminal domain from α1 to α2 of the *E. coli *zinc transporter YiiP can be superimposed onto the equivalent portion of human copper metallochaperone Hah1 with a root mean square deviation of 1.8 Å for 42 common C_α _positions, although there is no sequence homology between the C-terminal domain and Hah1 after a evolutionary period of more than thousand millions of years [[19], see ref. [21]], while the soluble fragment from *Thermus thermophilus *zinc transporter CzrB overlays the cytoplasmic domain of YiiP with a C_α_-root mean square deviation of 1.8 Å over 79 residues with an overall sequence identity of 8.8% [20].

**Figure 1:**
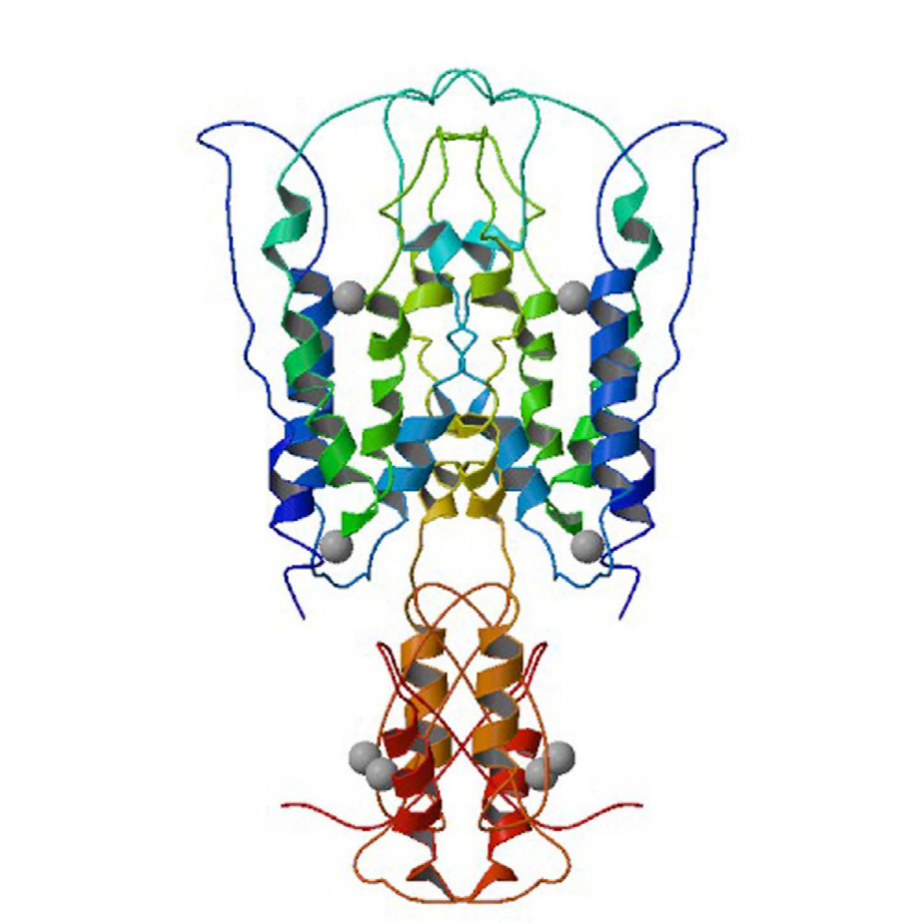
**Biological molecule image for the zinc transporter YiiP (17)**. The biological molecule consists of a pair of identical protein chains, each composed of (a) six transmembrane domains (blue and green) at the extracellular side, (b) a hydrophilic C-terminal domain with a αββαβ fold (red and orange) at the intracellular side, and (c) four zinc-populated sites (top-down designated as Z1 to Z4). Zinc ions are represented by gray spheres.

The zinc transporting function is attributed to the homologous hydrophobic domain, which is thought to be composed of a bundle of six transmembrane segments, denoted as TM1 to TM6, in an α-helical configuration, a structural theme found in many other membrane channels and transporters [21,22]. A representative member of the cation diffusion facilitator family is YiiP, a dimer of two identical 33-kD integral zinc transporter membrane proteins found in the plasma membrane of *E. coli *[23]. Recently, X-ray diffraction analysis of YiiP crystals and 12 heavy-atom derivative crystals revealed YiiP dimers at a resolution of 3.8 Å with individual subunits, each containing six bilayer-spanning α-helices, a C-terminal domain with an αββαβ fold, and four zinc-populated sites (Fig. 1) [19,24]. Most recently, the C-terminal domain of CzrB was overexpressed in *E. coli *and the crystal structure of the apo and zinc forms of the soluble fragment (CzrB_sf_) solved to 1.7 and 1.8 Å, respectively [20]. We have used this, in combination with the structure of YiiP, to examine the effects of the missense mutation on the 3D structure of the ZnT-8 protein complete with sensor, actuator and transporter parts.

## Methods

We retrieved information regarding the amino acid sequences of human ZnT-2 (Q9BRI3), ZnT-3 (Q99726), and ZnT-8 (Q8IWU4), mouse ZnT-8 (Q8BGG0), *E. coli *zinc transporters YiiP (Q1R404) and ZitB (Q8×400), *Thermus thermophilus *zinc transporter CzrB (Q8vlX7) and *Ralstonia metallidurans *zinc transporter CzcD (P13512) from the UniProtKB/Swiss-Prot databank [25]. The crystal structure of YiiP was retrieved from the Protein Data Bank [19,26]. C-terminal domains of *Homo sapiens *ZnT-8 and *E. coli *zinc transporter YiiP sequences were aligned and with a typical homology modeling exercise, the most probable 3D models were created with the program Modeller, version 9 [27]. The model was validated using the PSVS validation server [28-31] exclusive of the MolProbity clashscore because for this sort of analysis to be reliable, the reference crystal structure needs to be better than 2.0 Å resolution [32] (Table 2). All model representations were prepared using the program Pymol (DeLano Scientific) [33]. Secondary structure elements of the C-terminal domain of the zinc transporters CzrB were indicated as α-helices and β-pleated sheets according to the specifications of Cherezov *et al*. [20].

**Table 2:** Validation statistics of the homology model of the C-terminal domain of YiiP generated with the PSVS server [28].

Structure quality factors - overall statistics		
	**Mean score**	**Z-score^a^**
Procheck G-factor (phi/psi only) [29]	- 0.75	- 2.64
Procheck G-factor (all dihedral angles) [29]	- 0.49	- 2.90
Verify3D [30]	0.22	- 3.85
ProsaII (-ve) [31]	0.09	- 2.32
Ramachandran plot summary from Procheck [29]		
Most favoured regions	72.0%	
Additionally allowed regions	17.3%	
Generously allowed regions	8.0%	
Disallowed regions	2.7%	

^a^With respect to mean and standard deviation for a set of 252 X-ray structures < 500 residues, of resolution ≤ 1.80 Å, R-factor ≤ 0.25 and R-free ≤ 0.28; a positive value indicates a 'better' score.

## Results

**Figure 2:**
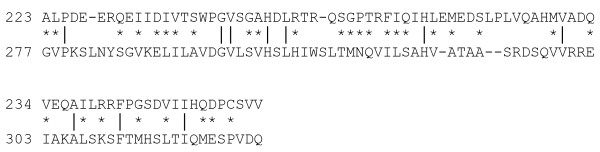
**Alignment of the human ZnT-8 (lower row) and *E. coli *zinc transporter YiiP (upper row) C-terminal domain sequences**. Amino acid residues are indicated by single letters. A vertical line connects identical residues and a * connects homologous residues.

We focused our analyses on the C-terminal domain of ZnT-8 because position 325, at which the mutation takes place, is located on this part of the protein. A model of the C-terminal domain of the human ZnT-8 protein was generated by homology modeling based on the known crystal structure of the *E. coli *zinc transporter YiiP at 3.8 Å resolution. The two sequences share 12.3% identical and 39.5% homologous residues (Fig. 2) resulting in an overall homology of 51.8%, which is well within the safe limits for homology modeling. A model was generated with Modeller [27] and validated using the PSVP validation server (Table 2) [28]. The generated models of the wild-type ZnT-8 protein and its Arg325Trp variant revealed a Y-shaped architecture consisting of a pair of identical protein chains, each composed of six transmembrane domains, a hydrophilic C-terminal domain with a αββαβ fold and four zinc receiving domains (designated as Z1 to Z4) (figure not shown). In our model, the Arg325 side-chain is at approximately 15 Å from the Z2 site.

**Figure 3:**
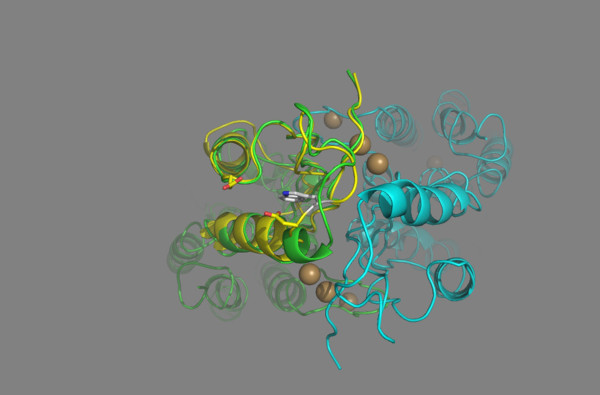
**In this diagram of the ZnT-8 R325W variant, the loop domain linking β2 to α2 at the outer edge of the C-terminal domain intracellular part faces the viewer**. Note that Trp325 points away from the interface with the other monomer. The bacterial structure is indicated in green, the human structure in yellow, and the other monomer in blue. The four front spheres at the interface of the intracellular domains represent the positioning of the two binuclear zinc ion clusters in sites Z3 and Z4 in each of the protomers, and the faint back spheres the zinc ion in site Z2 in each of the protomers. The side-chains of Trp325, Asp326, and Asp295 are presented by a "ribbon structure".

**Figure 4:**
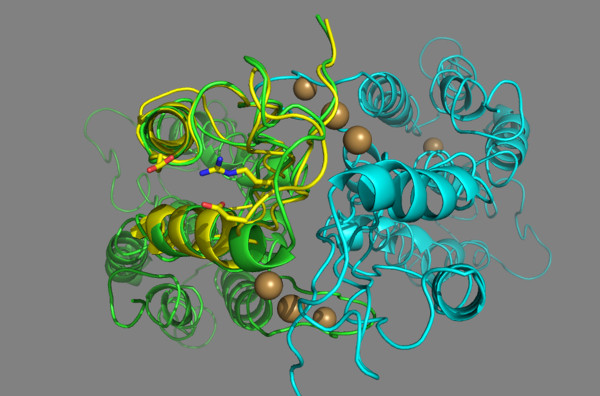
**Close-up view of the loop domain linking β2 to α2 (in front of the figure) at the outer edge of the C-terminal domain intracellular part of the 3D structure of the wild-type ZnT-8 protein**. Note that Arg325 points away from the interface with the other monomer. The bacterial structure is indicated in green, the human structure in yellow, and the other monomer in blue. The four front spheres at the interface of the intracellular domains represent the positioning of the two binuclear zinc ion clusters in sites Z3 and Z4 in each of the protomers, and the back spheres the zinc ion in site Z2 in each of the protomers. At the back of the figure, the faint sphere represents a zinc ion in site Z1. The model predicts salt bridges linking the ε-NH_3_^+ ^group of Arg325 and the β-COO^- ^group of Asp326 as well as Asp295. The side-chains of Arg325, Asp326, and Asp295 are presented by a "ribbon structure".

Fig. 3 displays the superposition of the loop connecting the second β-strand to the second α-helix of the C-terminal domain of the ZnT-8 R325W variant upon the structure of the YiiP zinc transporter. The α2-helix of the ZnT-8 mutated protein has Trp325 as the penultimate N-terminal residue. Further, the side-chain of Trp325 points away from the interface with the other monomer. In the wild-type ZnT-8 protein, the α2-helix has Arg325 as the penultimate N-terminal residue, while the Arg325 side-chain has an orientation virtually identical to that of Trp325 in the ZnT-8 R325W variant (Fig. 4). Arg325 may form an interchain noncovalent, electrostatic interaction (salt bridge) with its adjacent Asp326 and an intrachain salt bridge with Asp295 of the α1-helix of the C-terminal domain (Fig. 4). Table 3 shows the alignment of loop-domains linking the second β-strand to the second α-helix of the C-terminal domain of ZnT-8 and other ZnT proteins. The secondary structure elements (α-helix, β-strand, and loop) are indicated according to the 1.8 À resolution zinc-CzrB_sf _model [20]. Position 325, at which the mutation takes place, corresponds to a charged Arg residue. By replacing it with a Trp residue, the positive charge is removed, thereby affecting the electrostatic interactions with the protein.

**Table 3:** Sequence alignment of the loop domain linking β2 to α2 of YiiP and representative members of the cation diffusion facilitator family.

CDF homologs	Amino acid sequence	Residue number
	◀----β2---▶ ◀-L3-▶ ◀----- α2 --------	
YiiP	PTRFIQIHLE MEDSLP LVQAHMVADQVEQAI	286
CzrB	PRSFLEFHLV VRGDTP VEEAHRLCDELERAL	269
ZitB	EKPVMTLHVQ VI---P PHDHDALLDQIQHYL	275
CzcD	GKASLTVHVV NDTA-- VNPEMEVLPELKQML	273
msZnT-8	NQVILSVHVA TA---A SQDSQSVRTGIAQAL	337
hmZnT-2	AQPVLSVHIA IAQN-- -TDAQAVLKTASSRL	293
hmZnT-3	TYHVASAHLA IDST-- -ADPEAVLAEASSRL	357
hmZnT-8	NQVILSAHVA TA---A S**R**DSQVVRREIAKAL	339

The human ZnT-8 residue R325 is in boldface. YiiP and ZitB are bacterial CDF homologs from *Escherichia coli*, CzcD from *Ralstonia metallidurans*, CzrB from *Thermus thermophilus*, msZnT-8 from *Mus musculus *(mouse), and ZnT-2, ZnT-3, and ZnT-8 from *Homo Sapiens*.

## Discussion

This report is a description of a 3D atomic-resolution model of the C-terminal domain of a β-cell-specific Zn^2+ ^transporter, ZnT-8, predicted from the type 2 diabetes-associated gene variant SLC30A8 R325W. As for studies in zinc transporter functioning [20,34], we used homology modeling, which is based on the general observation that evolutionarily related (homologous) proteins are likely to have similar structures and currently gives the most accurate and reliable models [35]. We built structural models for ZnT-8 and its gene variant R325W, based on the known structure of a close homologue (template) YiiP, a membrane transporter that catalyzes Zn^2+^/H^+ ^exchange across the inner membrane of *E. coli*. The quality of the homology model is dependent of the quality of the sequence alignment and template structure [36]. Alignment of the ZnT-8 and YiiP C-terminal domain sequences revealed an overall homology of 51.8%, which is well within the safe limits for homology modeling (Fig. 2). The model had 89.3% residues in the allowed regions of the Ramachandran plot, a value consistent with a value of 90.1% reported for the total structure of ZnT-8 [37].

The YiiP protein shows the main characteristics of a family of ubiquitous zinc transporters termed cation diffusion facilitator including: (a) a homodimer held together in a parallel orientation through four zinc ions at the interface of the cytoplasmic domains, with the two transmembrane domains swung out to yield a Y-shaped structure; (b) a 33-kD integral transmembrane protein composed of a compact bundle of six transmembrane segments in an α-helical configuration (residues 1-211) arranged in a way that creates an extra- and an intracellular cavity on either side of the membrane to make room for one zinc ion, Z1 and Z2, respectively; and, finally, (c) a hydrophilic C-terminal domain (residues 212-300) consisting of three β-strands and two α-helices (α1-β1-β2-α2-β3 order). The latter exists as a three-stranded antiparallel β-sheet with the overall shape of a flattened ellipsoid. The rounded part of the domain is made up of the two α-helices laying on top of the β-sheets [38-40]. At the interface with the other monomer and close to the β-sheets, a binuclear zinc cluster is located at the sites Z3 and Z4 (Figs. 3 and 4). Finally, the ZnT-8 protein was predicted to adopt the same topology as the other ZnT proteins with six transmembrane helices [11]. Collectively, the data presented here suggest that the quality of the sequence alignment and template structure tend to be reliable for producing a 3D model of the ZnT-8 C-terminal domain to investigate the structural consequences of the substitution of Trp for Arg at position 325.

Recently, Cherezov *et al*. reported seminal data on the mechanism of transmembrane zinc transport via the putative zinc transporter CzrB [20]. Briefly, the proposed transport model suggests that in the absence of zinc the two flattened ellipsoids (αββαβ motif) as a dimer are splayed apart by charge repulsion. This will contribute to holding the two C-terminal domains apart and will serve to direct zinc ions into Z3 and Z4 as the intracellular concentrations of the ion rise. Thus, the cytosolic zinc concentration is sensed by the exposed C-terminal domain. When zinc binds to Z3 and Z4, a large conformational change occurs that closes the two ellipsoids (αββαβ motif) and hence enlarge the dimerization surface area from ~400Å in the absence of zinc to ~1050Å in its presence. The net effect of this zinc binding is to change the relative disposition of the cytosolic Z2 domain environment in such a way that a zinc-loaded metallochaperone can dock for delivery and transport of its zinc cargo to this intracellular cavity. The ion then travelers across the membrane into the extracellular cavity Z1 where effectively it is out of the cell.

Our homology model predicts that the Arg325 side-chain is located at approximately 15 Å from the compact bundle of six transmembrane helices. It is thus rather distant from the transmembrane domain site including the extra- and the intracellular cavity Z1 and Z2, respectively, which suggests that the Arg to Trp substitution at position 325 is unlikely to affect the protein folding near the cavity loci and the uploading of zinc.

The overall shape of the C-terminal domain is that of an flattened ellipsoid existing of three β-strands with the central strand running parallel and anti-parallel to its neighbor on either side and on top two α-helices. The three β-sheets create a planar and distinctly polar surface of the ellipsoid with a pronounced negative charge at the interface with the other monomer, the region with the bridged zinc ions Z3 and Z4 [20]. Our models predict that both the side chain of Arg325 and Trp325 point away from the interface with the other monomer (Fig. 3, 4). These data suggest that the amino acid residue at position 325 is neither involved in dimerization contacts, nor in the zinc coordination sites Z3 and Z4, and that the three β-sheets shield the positive charge of the ε-NH_3_^+ ^group of Arg325 from the sites Z3 and Z4 [[19], see Fig. S3; [20], see Fig. 3]. The existence of an positively charged Arg325 side chain on the outside of the ellipsoid pointing away from the interface with the other monomer in the presence a pronounced negative charge on the inner side of the ellipsoid reflects this effective way of shielding. The above data support the concept that the substitution of Trp for Arg at position 325 is unlikely to affect the protein folding of the exposed C-terminal domain, the sensor of the cytosolic zinc concentration.

Furthermore, our model of the wild-type ZnT-8 predicts an inter- and an intrachain salt bridge linking the ε-NH_3_^+ ^group of Arg325 to both the β-COO^- ^group of Asp326 and Asp295. Because the magnitude of the electrostatic force between two point electric charges is inversely proportional to the square of the total distance between the two charges, the magnitude of the electrostatic force between Arg325 and Asp295 is approximately 25% compared to the magnitude of the electrostatic force between Arg325 and Asp326. These observations suggest that the electrostatic interactions marginally contribute to the stabilization of the αββαβ fold of the C-terminal domain. A Trp325 would fit there, but the salt bridges would be lost, and this loss could alter the conformational state of the α1-helix and, in turn, the zinc binding and transport. Surprisingly, sequence alignment of wild-type ZnT-8 and YiiP revealed that Arg325 found in the former protein seems to be replaced by a Val residue in the latter protein, implying the disappearance of the salt bridges in the protein structure of YiiP (Table 2). For similar reasons, these salt bridges are absent in the prototypes of the bacterial members of the cation diffusion facilitator family homologs CzrB from *Thermus thermophilus*, ZitB from *E.Coli *and CzcD *from Ralstonia metallidurans*, and in mouse ZnT-8 (Table 2). A sequence alignment of the C-terminal domain from the ZnT-2, -3, and -8 proteins revealed that Arg325 in ZnT-8 was substituted by Thr279 in ZnT-2 and by Ala343 in ZnT-3 (Table 2). Additionally, an analysis of the phylogenetic tree for all identified human ZnT family proteins indicated that ZnT-2, -3, and -8 cluster closely together [19]. Thus, independent of the existence of the inter- and intrachain salt bridges, all currently known structures of metallochaperones and their target domains fully control the activity of intracellular zinc transport [39].

In contrast to our study on ZnT-8, Nicolson *et al*. reported that cells expressing the ZnT-8 Trp325 protein transport more zinc into cells, than cells expressing wild-type ZnT-8 Arg325 protein [37]. However, these observations are likely incorrect, because these conclusions appear to be based on an assay for ZnT-8 transporter activity revealing that the transporter may operate in the reverse direction whereas there are no ATPase domains in ZnT-8 which may be required for ZnT-8 to operate in the reverse direction. For instance, non-specific events may be responsible for the observed different rates of zinc transport, i.e., difference in the number of zinc transporters per area unit of secretory granules of β-cells expressing either wild-type or Trp325 variant of ZnT-8, or difference in background color of microscopy images of control cells versus β-cells expressing either wild-type or Trp325 variant of ZnT-8, as very similar experiments revealed a substantial amount of zinc in control cells in normal conditions versus ZnT-8-expressing cells, i.e., 830 ± 109 μg Zn/g protein and 1072 ± 78 μg Zn/g protein, respectively [12]. Nicolson *et al*. also concluded that the resultant introduction of positive charge into the region of monomer interface of ZnT-8, close to the predicted sites of bound structurally important zinc ions, may be expected to affect the kinetics of zinc transport [37]. Without any particular detail of a 3D model for ZnT-8, their conclusions are based merely on the observation that R325 resides at the monomer interface as our models predict that the side chains of both Arg325 and Trp325 point away from the interface with the other monomer. To resolve the above discussed differences and to show that wild-type Arg325 and Trp325 variant of ZnT-8 are no different in their ability to form dimers and their ability to transport zinc, we propose to improve the experimental conditions to prevent reverse zinc transport as described for ZnT-8 [37] and ZnT-5 [41]. Next, we propose the following sequence of experiments: (a) to assay directly the functional activity of ZnT-8, measure the transmembrane flux of zinc across the plasma membrane of *Xenopus laevis *oocytes [41]; (b) inject oocytes with wild-type Arg325 ZnT-8 RNA, with Trp325 variant of ZnT-8 RNA or water; (c) assay zinc uptake into oocytes by measuring ^65^Zn^2+ ^incorporation into single oocytes as γ emission [42].

According to the data presented by Cherezov et al. [20], Arg325 is a part of the α2 helical structure of the C-ternminal domain (Table 2). Arg has a helix-forming behavior and its tendency is to stabilize α-helices [43]. Also, Trp has a helix-forming behavior, which makes it unlikely that the R325W mutation affects the α2-helix folding.

An obvious limitation of our study is largely inherent to all theoretical models in biochemistry in general, which never allow complete certainty [36]. Our model of the ZnT-8 protein was based on a crystal structure of the *E. coli *zinc transporter YiiP. In the future, a high resolution ZnT-8 structure model will help to identify all specific residues that may contribute to full transport activity of zinc. Furthermore, we cannot fully exclude the possibility that the presence of a Trp residue at position 325 does not interfere with the binding of a putative zinc metallochaperone. However, cross-sectional analyses of 75-g 3-h oral glucose tolerance test results for a large sample of Caucasians and for populations with an exceptionally high prevalence of type 2 diabetes revealed inverted-U plots of insulin vs. glucose in plasma, implying direct proof of excellent insulin secretion during the pre-diabetic state, and consequently of adequate zinc transfer to the correct sites in the pancreatic islet cells [44-46].

## Conclusions

This modeling predicts that both Arg325 and Trp325, amino acids with a helix-forming behavior and penultimate N-terminal residues in the α2-helix of the C-terminal domain, are shielded by the planar surface of the three cytoplasmic β-strands and hence unable to affect the sensing capacity of the C-terminal domain. Moreover, the amino acid residue at position 325 is too far removed from the docking and transporter parts to affect their local protein conformations. These data suggest that the inherited R325W abnormality in SLC30A8 may be tolerated and results in adequate zinc transfer to the correct sites in the pancreatic islet cells and provide support to assume that the *SLC30A8 *gene variant R325W has a low predicted value for future type 2 diabetes at population-based level [7-9].

## Abbreviations

3D: three-dimensional; *E.coli*: *Escherichia coli*; *SLC30A8*: solute carrier family 30 zinc transporter member 8; SNP: single nucleotide polymorphism; ZnT: zinc transporter.

## Competing interests

The author declares that they have no competing interests.
